# The efficacy and safety of pramipexole ER versus IR in Chinese patients with Parkinson’s disease: a randomized, double-blind, double-dummy, parallel-group study

**DOI:** 10.1186/2047-9158-3-11

**Published:** 2014-06-02

**Authors:** Ying Wang, Shenggang Sun, Suiqiang Zhu, Chunfeng Liu, Yiming Liu, Qing Di, Huifang Shang, Yan Ren, Changhong Lu, Mark Forrest Gordon, Nolwenn Juhel, Shengdi Chen

**Affiliations:** 1 Ruijin Hospital affiliated to Shanghai Jiao Tong University School of Medicine, Shanghai, China; 2 Union Hospital affiliated to Tongji Medical College of Huazhong University of Science and Technology, Wuhan, China; 3 Tongji Hospital affiliated to Tongji Medical College of Huazhong University of Science and Technology, Wuhan, China; 4 The Second Affiliated Hospital of Soochow University, Suzhou, China; 5 Qilu Hospital affiliated to Shandong University, Jinan, China; 6 Nanjing Brain Hospital, Nanjing, China; 7 West China Hospital affiliated to Sichuan University, Chengdu, China; 8 First Affiliated Hospital of China Medical University, Shenyang, China; 9 Boehringer Ingelheim International Trading (Shanghai) Co, Ltd, Shanghai, China; 10 Boehringer Ingelheim Pharmaceuticals, Inc, Ridgefield, CT, USA; 11 Boehringer Ingelheim France S.A.S, Reims, France; 12 Department of Neurology, Ruijin Hospital affiliated to Shanghai Jiaotong University School of Medicine, Shanghai, China

**Keywords:** Parkinson’s disease, Pramipexole ER, Pramipexole IR, Non-inferiority, Unified Parkinson’s Disease Rating Scale (UPDRS), Safety

## Abstract

**Objective:**

To evaluate the non-inferiority of pramipexole extended-release (ER) versus immediate-release (IR) in Chinese patients with Parkinson’s disease (PD) in a double-blind, randomized, parallel-group study.

**Methods:**

Subjects were Chinese patients with idiopathic PD with diagnosis ≥ 2 years prior to trial, age ≥ 30 years old at diagnosis, and Modified Hoehn and Yahr score 2-4 during ‘on’-time. Subjects received treatment with pramipexole ER (n=234) or IR (n=239). Non-inferiority was based on the primary endpoint, the change from baseline to end of maintenance (week 18) in the UPDRS (Parts II + III) total score.

**Results:**

For the primary endpoint, the adjusted mean changes (standard error) of UPDRS Parts II + III at week 18 were -13.81 (0.655) and -13.05 (0.643) for ER and IR formulations, respectively, using ANCOVA adjusted for treatment and centre (fixed effect) and baseline (covariate). The adjusted mean between group difference was 0.8 for the 2-sided 95% CI (-1.047, 2.566). Since the lower limit of the 2-sided 95% CI (-1.047) for treatment difference was higher than the non-inferiority margin of -4, non-inferiority between pramipexole ER and IR was demonstrated. The incidence of adverse events (AEs) was 68.8% in the ER arm and 73.6% in the IR arm with few severe AEs (ER: 2.1%; IR: 3.8%).

**Conclusion:**

Based on the UPDRS II + III score, pramipexole ER was non-inferior to pramipexole IR. The safety profiles of pramipexole ER and IR were similar. These results were based on comparable mean daily doses and durations of treatment for both formulations.

## Introduction

Parkinson’s disease (PD), a chronic degenerative neurological disorder involving motor and non-motor dysfunctions, inflicts emotional, financial and social burdens on patients, their families, and their social network [1]. The prevalence in the United States is 1-2% in persons at least 65 years of age and 4-5% in persons over 85 years. The prevalence in China in those aged 65 years or older is similar (1.7%) [2].

Dopamine substitution with either L-dopa or dopamine agonists (DAs) remains the main therapy for PD patients [3]. However, prolonged treatment with L-dopa could lead to frequent, disabling motor complications [4]. To delay the occurrence of L-dopa-related motor complications, patients with early PD often receive DAs as monotherapy, while most advanced PD patients receive L-dopa with a DA to reduce existing motor complications. Pramipexole (immediate-release [IR] and extended-release [ER]) is a non-ergot DA with a high selectivity for the D_2_ subfamily of dopamine receptors, with preferential affinity for D_3_ receptors, contributing to its excellent efficacy and acceptable side effect profile [5-7]. The efficacy of pramipexole ER versus IR was demonstrated in two international, placebo-controlled, double-blind, pivotal studies in patients with early PD (Asians 36.5%) and advanced PD (Asians 49.7%) after 18 weeks of treatment, with the Unified Parkinson’s Disease Rating Scale (UPDRS) II and III total score as primary endpoint [8,9] and in a 12-week study in Japanese patients with PD [10].

An evaluation of pramipexole ER versus IR in Chinese patients with PD has not previously been undertaken. This study tested for non-inferiority of pramipexole ER versus IR in Chinese patients with PD who could be concomitantly treated with L-dopa.

## Patients and methods

### Patients

The study enrolled early and advanced PD patients from 20 centers across China (September 2010 - January 2012). Subjects were Chinese patients diagnosed with idiopathic PD for at least 2 years; age ≥ 30 years old at diagnosis, Modified Hoehn and Yahr (H&Y) score of 2 to 4 during ‘on’-time. In patients taking standard or controlled release L-dopa or L-dopa/entacapone, dose has to be optimized and stable for at least 4 weeks prior to the baseline visit. If patients had motor fluctuations while taking L-dopa, ‘off’-time at waking should be no more than 6 hours daily during 2 consecutive days before the baseline visit.

All procedures were performed with the understanding and written consent of the subjects and with the approval of institutional review boards at participating institutions (Appendix).

### Randomization and study intervention

Randomization was conducted by a validated system using a pseudo-random number generator, making treatment assignment reproducible but not predictable. Patients underwent randomization in blocks of four at a 1:1 ratio to receive oral pramipexole ER or IR tablets. Randomization was stratified by center. Throughout the study, the persons who administered the medications, the raters, and the patients were all blind to medication assignments.

The 18-week treatment course consisted of two phases: up-titration (<7 weeks) to achieve optimal treatment response and maintenance (11 weeks), followed by down-titration. Pramipexole ER doses ranging from 0.375 mg to 4.5 mg per day were identical to approved daily doses for IR. All patients received routine care. The trial permitted treatment with common anti-PD medications if administered at a stable dose for at least 4-weeks prior to enrollment and if no dose change was planned during the treatment phase. The trial prohibited use of DA or central dopaminergic antagonist within 4-weeks prior to enrollment.

### Clinical assessment

Investigators determined the Modified H&Y scale stages (0–5). PD symptoms were assessed by the UPDRS Part II (Activities of Daily Living (ADLs)) and III (Motor Examination) for both ‘off’ and ‘on’ periods [11,12]. A treatment response was a decrease of score by at least 20% from baseline. UPDRS II score was calculated as mean of the ‘off’-time and ‘on’-time for advanced PD patients and as on-time for early PD patients. UPDRS III score was only assessed for the ‘on’-time. The Mini-Mental State Examination (MMSE) was used [13]. Subjects self-evaluated the likelihood of dozing with Epworth Sleepiness Scale [14]. General status was evaluated by the Clinical Global Impressions of Improvement (CGI-I) scale and overall status by the Patient Global Impression of Improvement (PGI-I) scale (1 point: very much better; 7 points: very much worse in both scales). The response based on CGI or PGI-I was defined as a rating of at least ‘much better’ when comparing the past week to baseline assessment.

### Safety assessment

Vital signs and adverse events were monitored throughout the study. Safety assessments were based mainly on the occurrence, frequency, and severity of adverse events (AEs), but also included comprehensive indexes (e.g. physical examination, electrocardiography, ophthalmologic monitoring, and routine laboratories). Safety data were collected from baseline through end of down-titration. AEs were considered mild if they were easily tolerated, moderate if they interfered with usual activity, and severe if they were incapacitating or caused inability to work or to perform usual activities. AEs were considered serious if they resulted in death, were immediately life-threatening, resulted in persistent or significant disability/incapacity, required or prolonged patient hospitalization, were a congenital anomaly/birth defect, or were deemed serious for any other reason.

### Statistical analysis

Sample size calculations determined that 223 subjects were required in each pramipexole group to have 90% power, assuming a non-inferiority margin between ER and IR of -4 points, a standard deviation (SD) of 13, and testing with a one-sided 97.5% confidence interval (CI). Results were presented as 95% 2-sided confidence interval, to allow the calculation of the two sides of the CI. One-sided 97.5% CI & two-sided 95% CI will yield the same results and are equivalent. To allow for early dropouts, the estimated target sample size was 234 subjects per group. The sample allocation was 1:1.

The statistical analyses were pre-specified. The full analysis set (FAS) included all patients who were randomized to treatment, received at least one dose of study medication, and had provided both a baseline assessment and at least one post-baseline assessment for primary endpoint. The treated set (TS) included all patients who were dispensed study medication and were documented to have taken at least one dose. The per-protocol set (PPS) included all evaluable patients from the FAS who completed at least 14 weeks ± 3 days of active treatment and presented no major protocol violation.

Unless otherwise specified, all efficacy results were based on FAS, whereas for patients who withdrew or were lost to follow-up, the last observation carried forward approach (LOCF) was used. For all analyses, data were censored on the date of first intake of L-dopa as rescue medication for patients who had not been taking concomitant L-dopa at baseline. If used (e.g. for FAS analysis), the LOCF approach was applied after censoring.

The primary efficacy endpoint was the change from baseline to the end of the maintenance at week 18 in UPDRS II + III scores (0–160 points). Non-inferiority was based on comparison of lower bound of 2-sided 95% CI using a non-inferiority margin of -4 points and evaluated with an analysis of covariance (ANCOVA) adjusted for treatment and centre (fixed effects) and baseline (covariate).

Statistical analyses on secondary endpoints were designed to show superiority of pramipexole ER vs. IR. Secondary continuous parameters were analysed using an ANCOVA model with same effects as primary endpoint, but using the respective baseline values of the following continuous endpoints as covariate: the percentage and duration of ‘off’-time during waking hours (diary based, patients with advanced PD); the percentage and duration of ‘on’-time during waking hours, without dyskinesia or with non-troublesome dyskinesia, or with troublesome dyskinesia; and the UPDRS Part II and Part III scores calculated separately.

The Cochran-Mantel-Haenszel test adjusted for centre was applied to these binary parameters:

• The proportion of patients with at least a 20% improvement relative to baseline in the percentage ‘off’-time during waking hours (diary based, advanced patients),

• The responder rate (at least much better/improved) for CGI-I and PGI-I,

• The proportion of patients with at least a 20% improvement relative to baseline in the UPDRS II + III score, and,

• The proportion of patients requiring L-dopa supplementation during the study (for patients without concomitant L-dopa at baseline).

Descriptive statistics based on the TS were used to analyze safety data, demographic and baseline characteristics, concomitant diseases and medication, and treatment compliance. AE analyses were based on number of patients with AEs (not number of AEs) and presented as descriptive statistics. SAS 9.2 software package was used for all statistical analyses.

## Results

### Demographic, baseline and treatment characteristics of the study participants

As depicted on subject flowchart (Additional file 1: Figure S1), 524 patients were eligible. Finally, 475 patients were randomized to receive pramipexole ER (n = 236) or IR (n = 239) and 473 patients received the assigned treatment of ER (n = 234) or IR (n = 239). Demographics and baseline conditions were comparable between the two arms (Table 1). The mean age (SD) of the subjects was 62 (9.06) years and 39.5% of them were at least 65 years old. The study population comprised 63% male. Slightly more patients had early PD (53.9%) than advanced PD (46.1%). The mean (SD) UPDRS II + III score was 44.9 (16.51) with a majority of the patients in H&Y stage 2 to 3 for on-phase. The high missing rate of the H&Y stage for the ‘off’ phase for subjects taking ER (44.0%) and IR (42.7%) was largely related to those subjects with early PD in whom an ‘off’ phase H&Y stage was not recorded. The mean duration of PD (SD) was 4.96 (3.21) years.

**Table 1:** Demographic and clinical characteristics of the study participants at baseline

**Characteristic**	**Pramipexole ER**	**Pramipexole IR**	**Total**
	**n=236**	**n=239**	**n=475**
**Age, mean (±SD) years**	62.2(9.10)	61.8(9.03)	62.0(9.06)
<65	140(59.8)	146(61.1)	286(60.5)
≥65	94(40.2)	93(38.9)	187(39.5)
**Female sex, n (%)**	80(34.2)	95(39.7)	175(37.0)
**Body mass index, mean (±SD) kg/m**^**2**^	23.3(2.85)	23.3(3.06)	23.3(2.95)
**Hoehn and Yahr stage, n (%)**			
*On phase*			
2-3	226(96.6)	234(97.9)	460(97.3)
4-5	8(3.4)	5(2.1)	13(2.7)
*Off phase*			
2-3	113(48.3)	119(49.8)	232(49.0)
4-5	18(7.7)	18(7.5)	36(7.6)
Missing	103(44.0)	102(42.7)	205(43.3)
**UPDRS score, mean (±SD)**			
II	13.0(4.92)	13.0(5.97)	13.0(5.47)
III	31.7(12.09)	32.1(12.78)	31.9(12.43)
II+III	44.7(15.69)	45.1(17.31)	44.9(16.51)
**PD stage, n (%)**			
Early	122(52.1)	133(55.6)	255(53.9)
Advanced	112(47.9)	106(44.4)	218(46.1)
**Duration of disease, mean (±SD) years**	5.11(3.33)	4.82(3.09)	4.96(3.21)
2 to <5, n (%)	146(62.4)	153(64.0)	299(63.2)
≥5, n (%)	88(37.6)	86(36.0)	174(36.8)
**MMSE score, mean (±SD)**	28.4(1.61)	28.4(1.44)	28.4(1.52)
**ESS score, mean (±SD)**	5.0(3.81)	5.4(4.13)	5.2(3.97)

EES, Epworth sleepiness scale; MMSE, Mini Mental State Examination; PD, Parkinson’s disease; UPDRS, Unified Parkinson’s Disease Rating Scale.

The chi-square test was used for categorical variables, and one-way analysis of variance for continuous variables. There were no significant between-group differences in any baseline characteristics.

Previous and concomitant medications (Additional file 2: Figure S2) were generally balanced between treatment arms. Previous PD therapies were reported in 11.6% of patients, with most being L-dopa and its derivatives (6.1% of patients) and DAs (5.3% of patients). Concomitant anti-PD therapies were reported in 94.7% of patients. At baseline, 86% of patients were receiving L-dopa or its derivatives, with mean dose of 433.1 ± 204.9 mg. There were few patients (ER 5.1%, IR 7.9%) with add-on therapies for PD.

At final assessment, most patients (ER 72.6%; IR 69.9%) were receiving a low daily dose (0.375, 0.75, or 1.5 mg) of study drug (mean, ER: 1.5 mg; IR: 1.6 mg). The mean treatment compliance was very high in both treatment arms (ER 99.0%, IR 100.1%).

### Primary outcome

**Figure 1:**
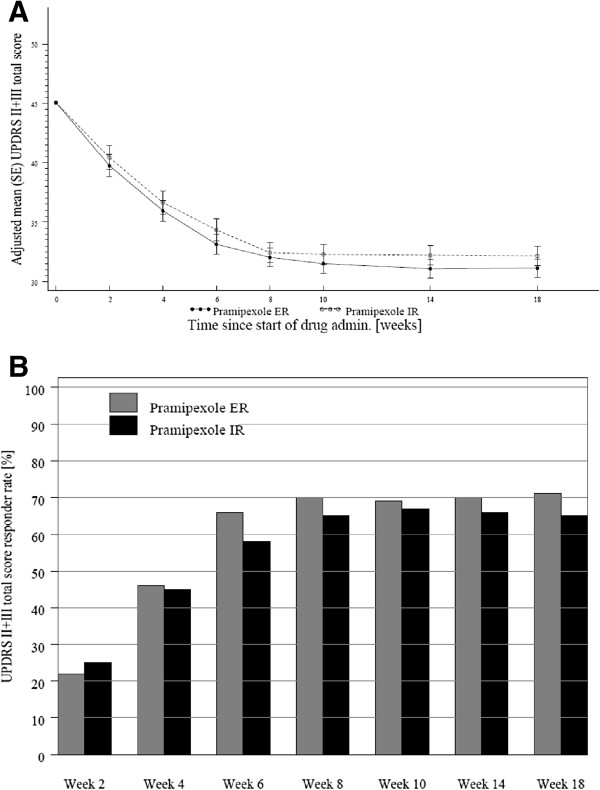
**Improvement in UPDRS II + III scores in both arms. (A)** Adjusted mean change (±SE) in UPDRS II + III score over time, full analysis set (FAS) (last observation carried forward, LOCF). Since the lower limit of the 2-sided confidence interval for treatment difference was higher than the non-inferiority margin, pramipexole ER was non-inferior to pramipexole IR. **(B)** Percentage of UPDRS II + III responder patients over time, FAS (LOCF). At week 18, the response rate was slightly higher in the ER arm than in the IR arm, but without a statistically significant difference between the arms.

The patients without UPDRS II + III score at baseline or during treatment were excluded, 228 patients in ER arm and 236 patients in IR arm were included in efficacy analysis. Both arms showed a similar improvement with most of improvement in UPDRS II + III scores after 6 weeks of treatment (Figure 1A). The mean (standard error) UPDRS II + III score at week 18 was 31.13 (0.97) for ER and 32.15 (1.04) for IR. For the primary endpoint, the adjusted mean changes (SE) of UPDRS Parts II + III at week 18 compared to baseline were -13.81 (0.655) and -13.05 (0.643) for ER and IR formulations, respectively, using ANCOVA adjusted for treatment and centre (fixed effect) and baseline (covariate). The adjusted mean between group difference was 0.8 for the 2-sided 95% CI (-1.047, 2.566). Since the lower limit of the 2-sided 95% CI (-1.047) for treatment difference was higher than the non-inferiority margin of -4, ER was shown to be non-inferior to IR. There was no statistical difference between the two arms at week 18 or at any other study visits.Responder patients for the UPDRS II + III score were those with a decrease of the score by at least 20% from baseline. The overall response rate for UPDRS II + III score steadily increased from 24.4% at week 2 to 67.7% at week 8 and thereafter stayed relatively constant (Figure 1B). At week 18, the response rate was slightly higher in ER arm (71.9%) than in IR arm (65.3%), but without a statistically significant difference.

### Secondary outcomes

**Figure 2:**
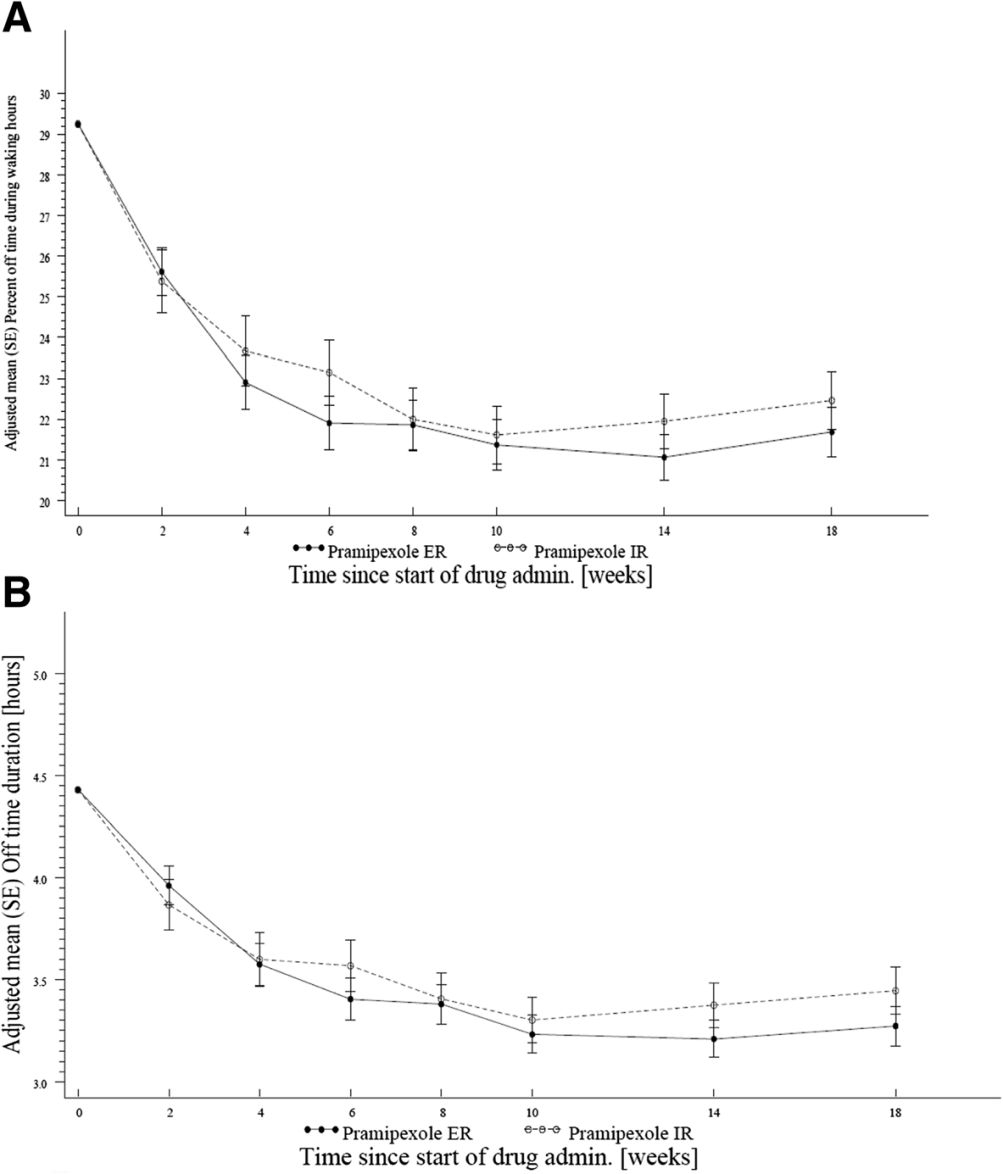
**Reduction of ‘off’-time in advanced PD patients. (A)** Adjusted mean change in the percentage of ‘off’-time over time in patients with advanced Parkinson disease (PD), full analysis set (FAS) (last observation carried forward, LOCF); **(B)** Adjusted mean duration (±SE) of ‘off’-time. For **A** and **B**: No difference was observed in the adjusted mean percentage ‘off’-time or duration between the two arms at any study visit (ANCOVA).

The mean (SE) ‘off’-time percentage during waking hours at baseline for advanced PD patients was 27.78 (0.81) for ER and 30.80 (1.21) for IR, and decreased to 21.68 (1.53) at week 18 for ER and 22.46 (1.61) for IR, respectively (no statistical difference for superiority). The adjusted mean change (ANCOVA) from baseline was -6.96 (1.51) for ER and -7.4 (1.54) for IR. The adjusted mean change (ANCOVA) from baseline in the mean duration of ‘off’-time was comparable between the two arms (ER: -1.0 (0.23) h; IR: -1.1 (0.23) h). Furthermore, the two arms revealed a similar response with most of the improvement having occurred after approximately 6 weeks of treatment (Figure 2). No difference was observed in the mean ‘off’-time percentage or duration between the two arms at any study visit (ANCOVA). Additionally, a similar response rate for ‘off’-time was observed in the ER and IR arms after 18 weeks (52.7% vs. 55.7%).

At study baseline, in advanced PD patients, on-time without dyskinesia comprised the majority of waking hours (ER: 67.98%; IR: 65.09%). The adjusted mean percentage/duration of on-time without dyskinesia increased in both groups: +7.0%/1.0 h (ER) and +4.3%/0.5 h (IR) (ANCOVA). The adjusted mean change (ANCOVA) from baseline in mean percentage of on-time without dyskinesia or with non-troublesome dyskinesia was 6.86 (1.57) for ER and 7.48 (1.60) for IR. The adjusted mean change (ANCOVA) from baseline in the mean duration of ‘on’-time without dyskinesia or with non-troublesome dyskinesia was +1.0 (0.26) h in both arms with no significant difference between the two arms at all study visits (ANCOVA) (Additional file 3: Figure S3). ‘On’-time with troublesome dyskinesia represented about 1% of waking hours reported by advanced PD patients, and only insignificant changes occurred during the treatment period in both arms.

The overall response rate for CGI-I increased from 41.2% at week 4 to 59.3% at week 8 and thereafter stayed relatively stable (Additional file 4: Figure S4A). At week 18, there were similar response rates for ER and IR (55.8% vs. 59.2%, no statistical difference). The overall response rate for PGI-I steadily increased from 12.1% at telephone call to 50.6% at week 6 and thereafter stayed relatively stable (Additional file 4: Figure S4B). At week 18, there were similar response rates for ER and IR (52.2% vs. 53.8%, no statistical difference).

In patients without concomitant L-dopa treatment at baseline, only 1 patient (ER arm) required L-dopa supplementation during study. As there was only one observation, the CMH test was not performed.

### Safety

The incidence of AEs was lower for ER (68.8%) than for IR (73.6%) (Table 2). The three most common AEs were somnolence (ER: 21.8%; IR: 14.6%), dizziness (ER: 12.8%; IR: 13.0%), and nausea (ER: 8.5%; IR: 7.1%). There were few severe AEs (ER: 2.1%; IR: 3.8%). The incidence of serious AEs was 2.6% for ER and 5.4% for IR. Incidences of drug-related AEs were similar (ER: 50.0%; IR: 52.7%). The three most common drug-related AEs were somnolence (ER: 18.8%; IR: 14.6%), dizziness (ER: 11.5%; IR: 12.1%), and nausea (ER: 7.3%; IR: 6.3%). Few AEs led to treatment discontinuation (ER: 4.7%; IR: 5.0%). A lower AE rate was observed in advanced PD patients treated with ER (67.9%) than those treated with IR (77.4%), while the rate was balanced for patients with early PD. AE System Organ Classes with an incidence rate of 10% or higher in either treatment arm were nervous system disorders (ER: 41.0%; IR: 36.8%), gastrointestinal disorders (ER: 25.6%; IR: 24.3%), and eye disorders (ER: 11.1%; IR: 13.0%). Of the treatment emergent AEs of eye disorders, blurred vision (ER: 3.4%, IR: 1.7%) and cataracts (ER: 2.1%, IR: 1.3%) occurred most frequently. The incidence of orthostatic hypotension was low in both arms (ER: 6.8%; IR: 5.1%), with about 1/3 of all cases being symptomatic.

**Table 2:** Treatment emergent adverse effects in the study participants

**Characteristic**	**Pramipexole ER**	**Pramipexole IR**	**Total**
	**n=234**	**n=239**	**n=473**
**Patients with any AE**	161(68.8)	176(73.6)	337(71.2)
Severe AEs	5(2.1)	9(3.8)	14(3.0)
Drug-related AEs	117(50)	126(52.7)	243(51.4)
AEs leading to discontinuation	11(4.7)	12(5.0)	23(4.9)
Serious AEs	6(2.6)	13(5.4)	19(4.0)
**Nervous system disorders**	96(41.0)	88(36.8)	184(38.9)
Somnolence	51(21.8)	35(14.6)	86(18.2)
Dizziness	30(12.8)	31(13.0)	61(12.9)
Dyskinesia	12(5.1)	18(7.5)	30(6.3)
Tremor	4(1.7)	6(2.5)	10(2.1)
PD	3(1.3)	9(3.8)	12(2.5)
**Gastrointestinal disorders**	60(25.6)	58(24.3)	108(22.8)
Nausea	20(8.5)	17(7.1)	37(7.8)
Constipation	17(7.3)	20(8.4)	37(7.8)
Abdominal discomfort	3(1.3)	7(2.9)	10(2.1)
Upper abdominal pain	6(2.6)	6(2.5)	12(2.5)
Vomiting	5(2.1)	5(2.1)	10(2.1)
**Eye disorders**	11(4.7)	8(3.3)	19(4.0)
**Vascular disorders**	8(3.4)	11(4.6)	19(4.0)
Orthostatic hypotension	5(2.1)	4(1.7)	9(1.9)
Hypotension	1(0.4)	5(2.1)	6(1.3)

## Discussion

Although prior studies demonstrated the non-inferiority of pramipexole ER versus IR, as reflected by improvement in UPDRS II and III scores after 18 weeks [8,9], no study of ER versus IR had been undertaken in Chinese patients with early and advanced PD. In this randomized, double-blind, double dummy, parallel-group study, ADLs of PD patients were improved by pramipexole ER, as evidenced by improvement in UPDRS Part II score assessed in the ‘off’ and ‘on’ periods, and mean of the ‘off’/‘on’ periods. This study showed similar improvements in motor function, as reflected by changes in UPDRS Part III score. ER improved UPDRS II + III total scores at week 18 with most of the improvement having occurred after 6 weeks of treatment. Analysis of non-inferiority indicated that ER was non-inferior to IR in improving ADLs and motor function of PD patients. Both arms showed similar overall response rates assessed by UPDRS II + III score. Analyses of secondary endpoints confirmed the findings for primary endpoint, showing similar results for both treatments. Exploratory comparisons revealed no difference between the two treatments for secondary endpoints, with the exception of the change in on-time with non-troublesome dyskinesia during waking hours, where the results favored IR.

Pramipexole ER and IR similarly reduced the ‘off’ period and increased the ‘on’ period without dyskinesia or with non-troublesome dyskinesia. Similar response rates for CGI-I and PGI-I were observed for ER and IR. These findings established the efficacy of pramipexole ER and its non-inferiority to IR. These findings are consistent with the results of the pivotal studies in patients with early and advanced PD [8,9].

An earlier study of pramipexole in Chinese patients with early or advanced PD in Hong Kong and Taiwan showed that pramipexole is safe and well-tolerated [15]. We found similar safety profiles of ER and IR tablets with no unexpected safety risks. This finding is consistent with an early study [16] showing no significant difference in AE profiles between the two pramipexole formulations. The three most common drug-related AEs in our study were somnolence, dizziness, and nausea. These may not be drug-specific as a similar rate of AEs has been reported for pramipexole ER and the placebo [10]. As in earlier studies [17], hypotension was seen in a very small proportion of our patients.

In conclusion, pramipexole ER is non-inferior to pramipexole IR based on the UPDRS II + III score at week 18. Both formulations are safe and well-tolerated by the patients and are effective for early and advanced PD in Chinese patients. These results are based on comparable mean daily doses and durations of treatment for both formulations.

## Appendix

### List of institutions and investigators participating in the study

Ruijin Hospital affiliated to Shanghai Jiao Tong University School of Medicine, Shengdi Chen, Ying Wang; Union Hospital affiliated to Tongji Medical College of Huazhong University of Science and Technology, Shenggang Sun; Tongji Hospital affiliated to Tongji Medical College of Huazhong University of Science and Technology, Suiqiang Zhu; The Second Affiliated Hospital of Soochow University, Chunfeng Liu; Qilu Hospital affiliated to Shandong University, Yiming Liu; Nanjing Brain Hospital, Qing Di; West China Hospital affiliated to Sichuan University, Huifang Shang; First Affiliated Hospital of China Medical University, Yan Ren; Zhongshan Hospital affiliated to Fudan University, Wei Fan; Huashan Hospital affiliated to Fudan University, Jian Wang; The First Affiliated Hospital of Chongqing Medical University, Guoguang Peng; Guangzhou First People’s Hospital, Xiaoping Pan; Peking University First Hospital, Xiangru Sun; Beijing Tiantan Hospital affiliated to Capital Medical University, Tao Feng; Southwest Hospital, Shugui Shi; Peking University Third Hospital, Dongsheng Fan; Peking Union Medical College Hospital, Zhenxin, Zhang; First Affiliated Hospital of Sun Yat-sen University, Jinsheng Zeng; The Second Affiliated Hospital of College of Medicine, Zhejiang University, Baorong Zhang; Sir Run Run Shaw Hospital, School of Medicine, Zhejiang University, Xingyue Hu.

### Full financial disclosures of all authors for the past year

1. Ying Wang participated in clinical trials sponsored by GSK, Eisai, Lundbeck, Novartis.

2. Shenggang Sun participated in clinical trials sponsored by Novartis, Servier, Eisai, GSK, Lundbeck.

3. Suiqiang Zhu participated in clinical trial sponsored by UCB.

4. Chunfeng Liu participated in clinical trials sponsored by Pfizer, UCB, GSK.

5. Yiming Liu participated in clinical trial sponsored by UCB.

6. Qing Di participated in clinical trial sponsored by Pfizer.

7. Huifang Shang paticipated in clincial trials sponsored by GSK, UCB.

8. Yan Ren has nothing to disclose.

9. Shengdi Chen participated in clinical trials sponsored by Novartis, Lundbeck, Eisai, Xian Janssen.

The above authors all served as investigators in this clinical trial sponsored by Boehringer Ingelheim International GmbH.

The above authors deny other interest relationships with industry.

10. Changhong Lu is an employee of Boehringer Ingelheim International Trading (Shanghai) Co, Ltd., China.

11. Nolwenn Juhel is an employee of Boehringer Ingelheim France S.A.S., Reims, France.

12. Dr. Gordon is an employee of Boehringer Ingelheim Pharmaceuticals, Inc., Ridgefield, CT.

## Competing interests

The authors declare that they have no competing interests.

## Authors’ contributions

YW: conception and design; acquisition of data; analysis and interpretation of data; co-authorship of first draft; critical revision of subsequent drafts. SS, SZ, CL, YL, QD, HS, YR: conception and design; acquisition of data; analysis and interpretation of data; critical revision of several drafts. CL: conception and design; acquisition of data; analysis and interpretation of data; administrative, technical, or material support; supervision; critical revision of several drafts. MFG: conception and design; acquisition of data; analysis and interpretation of data; administrative, technical, or material support; supervision; critical revision of several drafts of the submitted publication material based on review of coauthors. NJ: acquisition of data; analysis and interpretation of data; critical revision of the submitted publication material; and statistical expertise. SC: principle investigator and the national investigator; conception and design; acquisition of data; analysis and interpretation of data; authorship of first draft; and critical revision of subsequent drafts. All authors read and approved the final manuscript.

## Supplementary Material

Additional file 1: Figure S1 The subject flow chart. Click here for file

Additional file 2: Figure S2 Previous, baseline, concomitant and added antiparkinsonian therapies for the study participants. Click here for file

Additional file 3: Figure S3 (A) Adjusted mean (±SE) percentage of on-time without dyskinesia or with non-troublesome dyskinesia. (B) Adjusted mean (±SE) duration of on-time without dyskinesia or with non-troublesome dyskinesia. Click here for file

Additional file 4: Figure S4 (A) Percentage of CGI-I responder patients over time, FAS (LOCF). (B) Percentage of PGI-I responder patients over time, FAS (LOCF). Click here for file

## References

[B1] Olesen J Gustavsson A Svensson M Wittchen HU Jönsson B The economic cost of brain disorders in Europe Eur J Neurol 2012 19 1 155 162 22175760 10.1111/j.1468-1331.2011.03590.x

[B2] Tian YY Tang CJ Wu J Zhou JS Parkinson’s disease in China Neurol Sci 2011 32 1 23 30 21174138 10.1007/s10072-010-0461-8

[B3] Müller T Drug therapy in patients with Parkinson’s disease Transl Neurodegeneration 2012 1 10 10.1186/2047-9158-1-10 PMC3514092 23211041

[B4] Rascol O Goetz C Koller W Poewe W Sampaio C Treatment interventions for Parkinson’s disease: an evidence based assessment Lancet 2002 359 9317 1589 1598 12047983 10.1016/S0140-6736(02)08520-3

[B5] Pinter MM Pogarell O Oertel WH Efficacy, safety, and tolerance of the non-ergoline dopamine agonist pramipexole in the treatment of advanced Parkinson’s disease: a double blind, placebo controlled, randomised, multicentre study J Neurol Neurosurg Psychiatry 1999 66 4 436 441 10201413 10.1136/jnnp.66.4.436 PMC1736320

[B6] Parkinson Study Group Safety and efficacy of pramipexole in early Parkinson disease. A randomized dose-ranging study JAMA 1997 278 2 125 130 9214527 10.1001/jama.1997.03550020057038

[B7] Parkinson Study Group Pramipexole vs levodopa as initial treatment for Parkinson disease: a randomized controlled trial. Parkinson Study Group JAMA 2000 284 15 1931 1938 11035889 10.1001/jama.284.15.1931

[B8] Hauser RA Schapira AH Rascol O Barone P Mizuno Y Salin L Haaksma M Juhel N Poewe W Randomized, double-blind, multicenter evaluation of pramipexole extended release once daily in early Parkinson’s disease Mov Disord 2010 15;25 15 2542 2549 10.1002/mds.23317 20669317

[B9] Schapira AH Barone P Hauser RA Mizuno Y Rascol O Busse M Salin L Juhel N Poewe W Pramipexole ER Studies Group Extended-release pramipexole in advanced Parkinson disease: a randomized controlled trial Neurology 2011 23;77 8 767 774 10.1212/WNL.0b013e31822affdb 21832216

[B10] Mizuno Y Yamamoto M Kuno S Hasegawa K Hattori N Kagimura T Sarashina A Rascol O Schapira AH Barone P Hauser RA Poewe W Pramipexole ER Study Group Efficacy and safety of extended- versus immediate-release pramipexole in Japanese patients with advanced and (L)-dopa-undertreated Parkinson disease: a double-blind, randomized trial Clin Neuropharmacol 2012 35 4 174 181 22801294 10.1097/WNF.0b013e31825f77b9

[B11] Lang AE Koller WC, Paulsen G Clinical rating scales and video- tape analysis Therapy of Parkinson’s Disease 1990 New York: Marcel Dekker 3 30

[B12] Hoehn MM Yahr MD Parkinsonism: onset, progression, and mortality Neurology 1967 17 5 427 442 6067254 10.1212/wnl.17.5.427

[B13] Folstein MF Folstein SE McHugh PR Mini-mental state. A practical method for grading the cognitive state of patients for the clinician J Psychiatr Res 1975 12 3 189 198 1202204 10.1016/0022-3956(75)90026-6

[B14] Johns MW A new method for measuring daytime sleepiness: the Epworth sleepiness scale Sleep 1991 14 6 540 545 1798888 10.1093/sleep/14.6.540

[B15] Wong KS Lu CS Shan DE Yang CC Tsoi TH Mok V Efficacy, safety, and tolerability of pramipexole in untreated and levodopa-treated patients with Parkinson’s disease J Neurol Sci 2003 216 1 81 87 14607306 10.1016/s0022-510x(03)00217-x

[B16] Poewe W Rascol O Barone P Hauser RA Mizuno Y Haaksma M Salin L Juhel N Schapira AH Pramipexole ER Studies Group Extended-release pramipexole in early Parkinson disease: a 33-week randomized controlled trial Neurology 2011 77 8 759 766 21832218 10.1212/WNL.0b013e31822affb0

[B17] Hubble JP Koller WC Cutler NR Sramek JJ Friedman J Goetz C Ranhosky A Korts D Elvin A Pramipexole in patients with early Parkinson’s disease Clin Neuropharmacol 1995 18 4 338 347 8665547 10.1097/00002826-199508000-00006

