# Supplementary figures and images for: The efficacy and safety of pramipexole ER versus IR in Chinese patients with Parkinson’s disease: a randomized, double-blind, double-dummy, parallel-group study
Source: Transl Neurodegener. 2014 Jun 2;3:11. doi: 10.1186/2047-9158-3-11 (PMC4128609; doi:10.1186/2047-9158-3-11)

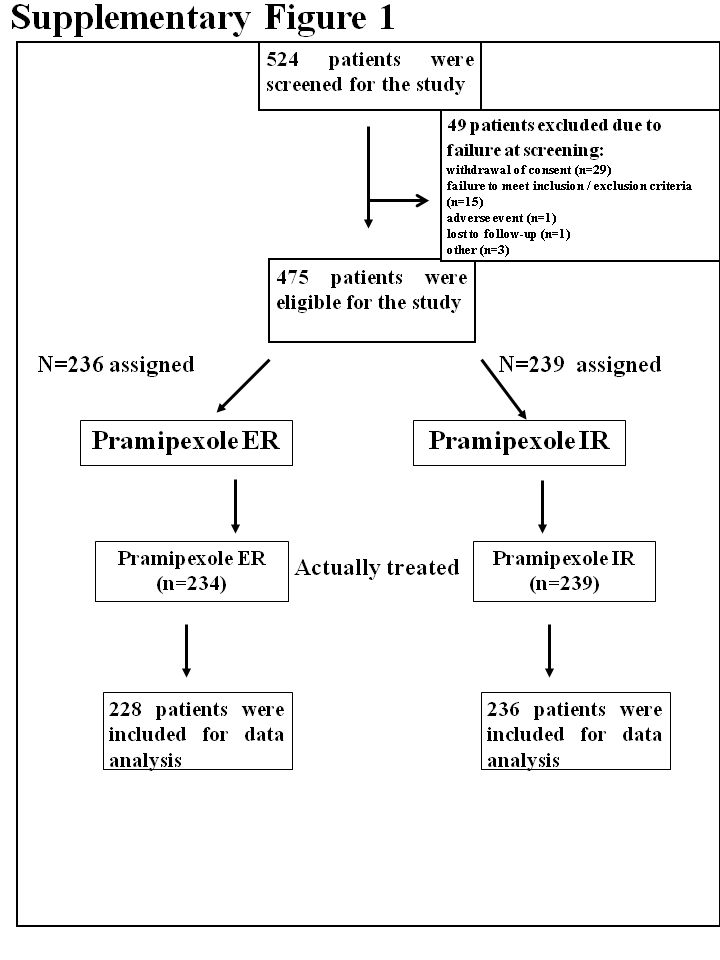

Supplement: Additional file 1: Figure S1 — The subject flow chart. [file 2047-9158-3-11-S1.tiff]

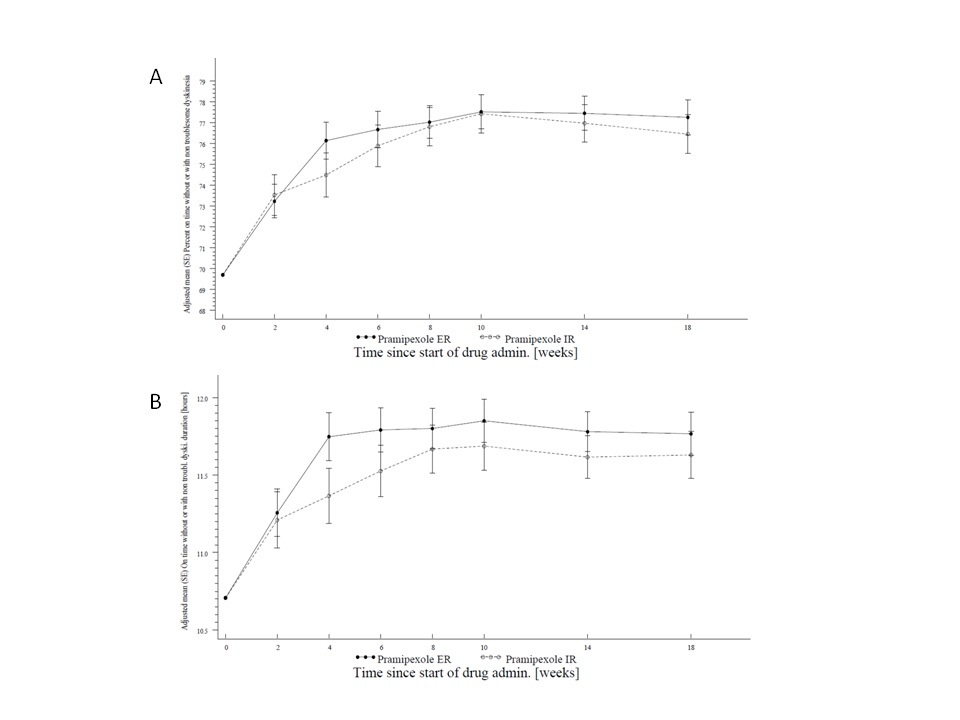

Supplement: Additional file 3: Figure S3 — (A) Adjusted mean (±SE) percentage of on-time without dyskinesia or with non-troublesome dyskinesia. (B) Adjusted mean (±SE) duration of on-time without dyskinesia or with non-troublesome dyskinesia. [file 2047-9158-3-11-S3.tiff]

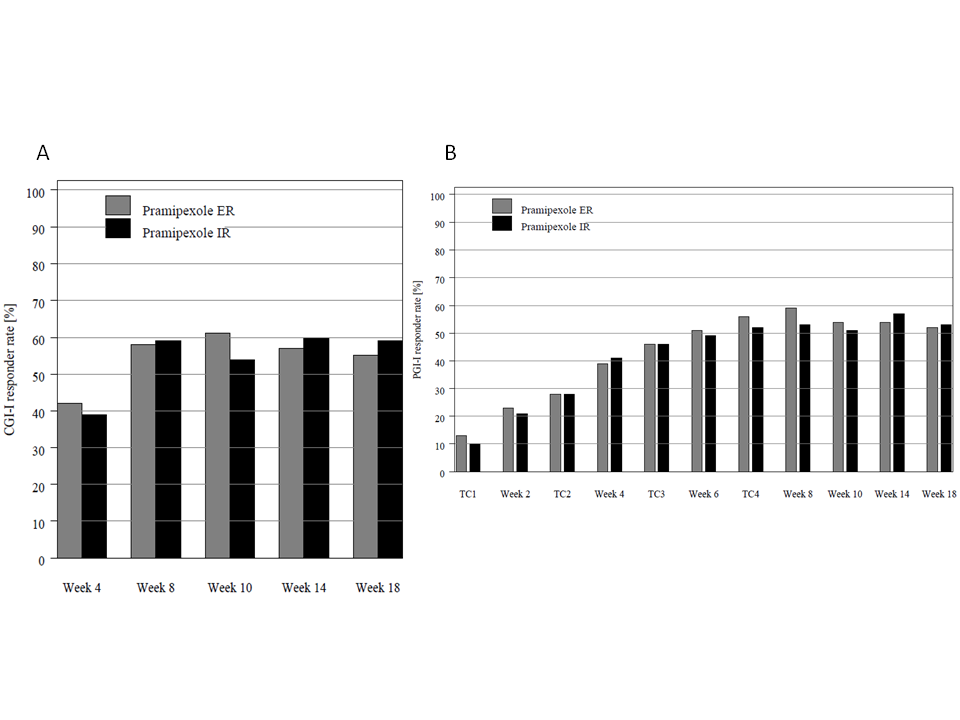

Supplement: Additional file 4: Figure S4 — (A) Percentage of CGI-I responder patients over time, FAS (LOCF). (B) Percentage of PGI-I responder patients over time, FAS (LOCF). [file 2047-9158-3-11-S4.tiff]
