# Supplementary material for: The efficacy and safety of pramipexole ER versus IR in Chinese patients with Parkinson’s disease: a randomized, double-blind, double-dummy, parallel-group study
Source: Transl Neurodegener. 2014 Jun 2;3:11. doi: 10.1186/2047-9158-3-11 (PMC4128609; doi:10.1186/2047-9158-3-11)
Supplement: Additional file 2: Figure S2 — Previous, baseline, concomitant and added antiparkinsonian therapies for the study participants. [file 2047-9158-3-11-S2.docx]

Supplementary Figure 2: Previous, baseline, concomitant and added antiparkinsonian therapies for the study participants

| **Characteristic** | **Pramipexole ER**  n=234 | **Pramipexole IR**  n=239 | **Total**  n=473 |
| --- | --- | --- | --- |
| **Prior antiparkinsonian medications, n(%)** | 30(12.8) | 25(10.5) | 55(11.6) |
| *L*-dopa or derivatives | 17(7.3) | 12(5.0) | 29(6.1) |
| Dopamine agonists | 13(5.6) | 12(5.0) | 25(5.3) |
| Others | 3(1.3) | 6(2.6) | 9(1.9) |
| **Baseline *L*-dopa therapy, n(%)** | 228(97.4) | 226(94.6) | 454(96.0) |
| Mean dose(±SD) mg | 442.73(202.21) | 423.12(201.32) | 433.12(204.85) |
| **Concomitant antiparkinsonian therapy, n(%)** | 224(95.7) | 224(93.7) | 448(94.7) |
| *L*-dopa or derivatives | 208(88.9) | 199(83.3) | 407(86.0) |
| Adamantane derivatives | 64(27.4) | 75(31.4) | 139(29.4) |
| Monoamine oxidase-B inhibitors | 35(15.0) | 35(14.6) | 70(14.8) |
| Others* | 22(9.4) | 18(7.2) | 37(8.4) |
| **Added antiparkinsonian therapy, n(%)** | 12(5.1) | 19(7.9) | 31(6.6) |
| *L*-dopa or derivatives | 12(5.1) | 18(7.5) | 30(6.3) |
| Others | 3(1.3) | 4(1.6) | 7(1.6) |

Prior antiparkinsonian medications refers to those with a stop date before Visit 2.

Concomitant antiparkinsonian therapy refers to therapy starting before the end of treatment and ending after first intake of the study drug.

Added antiparkinsonian therapy refers to therapy with a start date between first intake of the study drug and the final assessment.

*anticholinergics and selective beta blocking agents
